# Submaximal walking increases MicroRNA-126 and angiogenic markers in men and women with peripheral arterial disease

**DOI:** 10.1007/s00421-026-06360-0

**Published:** 2026-07-21

**Authors:** Amauri Maroto, Tiago Fernandes, Bruno R. A. Pelozin, Anthony S. Leicht, Nelson Wolosker, Luan Morais Azevêdo, Luiz Augusto Riani Costa, Paulo Rizzo Ramires, Cláudia Lúcia de Moraes Forjaz, Natan Daniel da Silva Junior

**Affiliations:** 1 Exercise Hemodynamic Laboratory, School of Physical Education and Sport, University of São Paulo, São Paulo, Brazil; 2 Laboratory of the Biochemistry and Molecular Biology of Exercise, School of Physical Education and Sport, University of São Paulo, São Paulo, Brazil; 3 Sport & Exercise Science, James Cook University, Townsville, Australia; 4 Albert Einstein Hospital, São Paulo, Brazil; 5 Ibirapuera University , São Paulo, Brazil

**Keywords:** Intermittent claudication, Physical exercise, MicroRNA, VEGF

## Abstract

**Purpose:**

Vascular dysfunction is a pathophysiological mechanism in peripheral artery disease (PAD). PAD patients exhibit reduced circulating expression of microRNA-126 (c-miRNA-126) and VEGF. Submaximal walking (SW) is recommended in PAD because it increases walking capacity, partially by improving vascular regulation, which can be observed after a walking session. Nevertheless, previous studies were conducted with maximal walking and in men. As women with PAD present worse disease characteristics and greater ischemia, they may be less responsive to SW, which needs to be investigated. Thus, this study compared the acute effects of SW on vascular function, c-miRNA-126 and angiogenic markers in men and women with PAD.

**Methods:**

Thirty patients (15 women) underwent, in a random order, 2 sessions: control (standing on treadmill) and SW (15 bouts of 2-min walking at moderate intensity intersected by 2-min rest). Circulating gene expression of miRNA-126, SPRED-1, PI3KR2, VEGF, and eNOS were evaluated before and after the interventions.

**Results:**

Both genders presented similar maximal walking capacity. Vascular function did not change after the interventions in either gender. After SW, both groups presented a similar decrease in PI3KR2 and similar increases in c-miRNA-126, VEGF and eNOS.

**Conclusion:**

In men and women with PAD, an acute SW similarly increases c-miRNA-126 expression, which inhibits PI3KR2, enabling the progression of the VEGF angiogenic signaling pathway of PI3K-AKT-eNOS. These results support that SW is equally effective in inducing molecular angiogenic changes in men and women with PAD, supporting its recommendation for both genders. Future studies should address these possible chronic effects of walking.

**Graphical abstract:**

**Figure:**
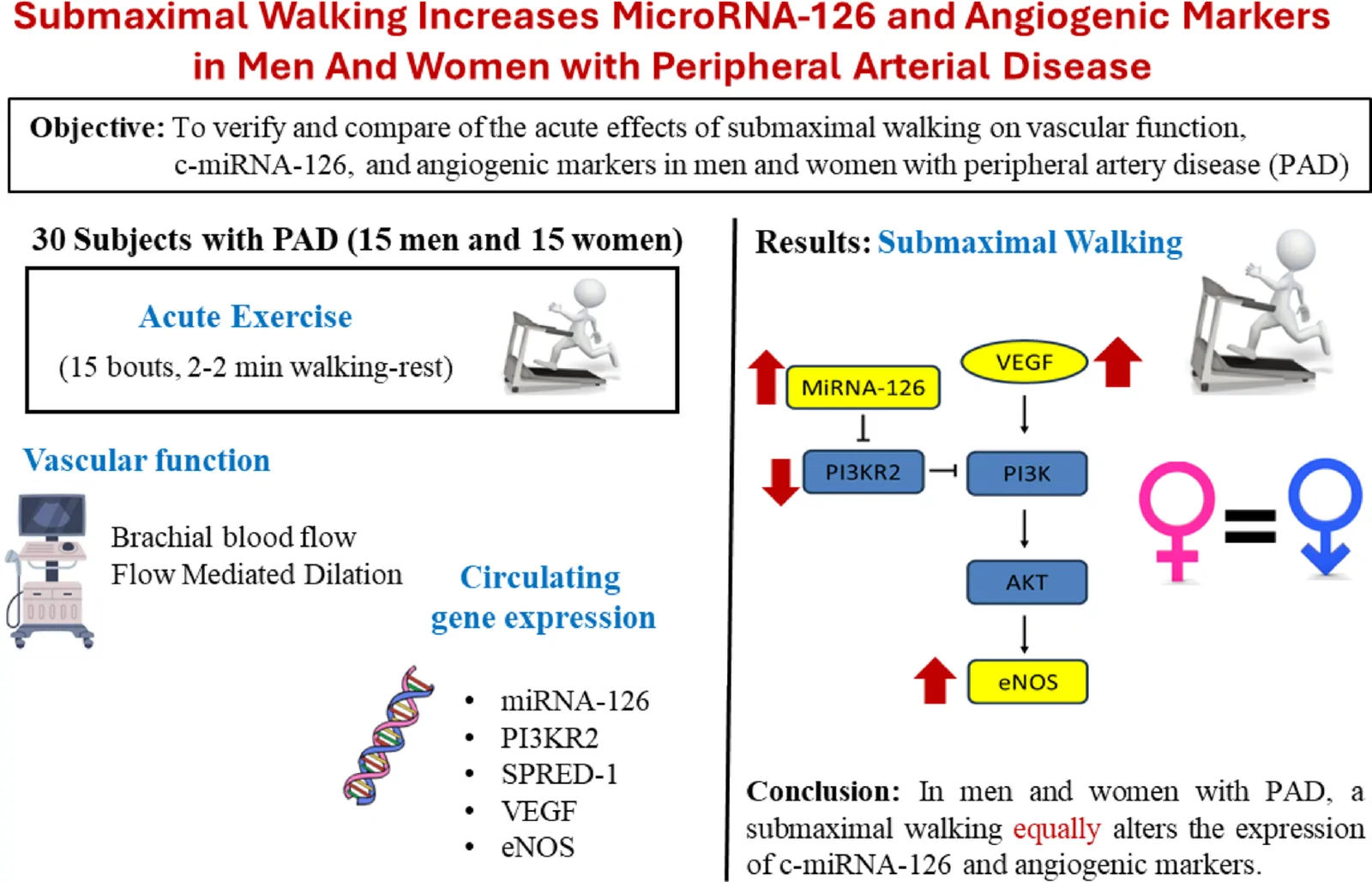

## Introduction

Peripheral artery disease (PAD) affects 3 to 10% of the adults worldwide(Kroger et al. 2006). Its prevalence increases with age (Norgren et al. 2007; Criqui et al. 2005), being similar or higher in women compared to men at advanced ages (Song et al. 2019). Intermittent claudication (IC), the leading symptom of PAD, is present in around 30% of the patients (Becker et al. 2002; Norgren et al. 2007) and is more frequent in men than women (Sigvant et al. 2007).

Due to obstruction of the arteries of the lower limbs and IC-related pain during walking, the functional capacity of the patients with PAD and IC is significantly reduced (Myers et al. 2008), decreasing their quality of life (Breek et al. 2001). Furthermore, the atherosclerotic disease present in PAD increases cardiovascular risk, resulting in high cardiovascular morbidity and mortality (Harris et al. 1995; Boger et al. 1997). In addition, these consequences of PAD and IC seem to be more severe in women than men (Hirsch et al. 2006). Therefore, the aim of PAD´s treatment is to increase functional capacity, reduce IC symptoms, improve quality of life and ameliorate cardiovascular prognosis, which should be primarily achieved through regular submaximal-intensity walking (Hirsch et al. 2006; Gerhard-Herman et al. 2017).

A central pathophysiological mechanism of PAD is vascular dysfunction (Vita and Hamburg 2010) evidenced by reduced flow-mediated dilation (FMD) (Harris et al. 1995), vascular endothelium growth factor (VEGF) (Palmer-Kazen et al. 2004) and muscle capillarity (Askew et al. 2005; Clyne et al. 1985). It is known that miRNA-126 that modulates the VEGF pathway is also decreased in PAD (Stather et al. 2013), which may partially explain the attenuated vascular response. MicroRNA-126 (miRNA-126) acts by inhibiting two negative regulators of the VEGF signaling pathway: sprouty-related protein 1 (SPRED-1) that inhibits the angiogenic pathway formed by RAF1-MEK-ERK; and phosphoinositol-2 kinase regulatory subunit 2 (PI3KR2) that inhibits the angiogenic pathway formed by PI3K-AKT-eNOS PI3KR2 (Fish et al. 2008).

In a previous study, we showed that an acute bout of maximal walking (i.e., a treadmill test conducted with a specific protocol for this population and limited by maximal claudication pain) increased c-miRNA-126 and VEGF levels in male patients with PAD (da Silva et al. 2015). Nevertheless, it is necessary to confirm whether the same vascular and angiogenic responses can be obtained with submaximal walking since this is the exercise training protocol recommended and used in clinical practice for patients with PAD (Gerhard-Herman et al. 2017). Furthermore, it is also important to investigate whether these responses can also be obtained in women.

Previous literature showed that female patients with PAD and IC have impaired microvascular function in the gastrocnemius (Gardner et al. 2010), presenting greater ischemia (Gardner 2002; Gommans et al. 2015) and lower oxygen saturation (Gardner et al. 2009) during exercise compared with men. These differences suggest that women with PAD may be less responsive to walking-induced vascular adaptations and may exhibit lower walking capacity than men. Nevertheless, in a previous study, we did not observe any differences in walking capacity and postexercise cardiovascular responses between men and women with PAD and similar clinical characteristics (Miyasato et al. 2021). One possible explanation is that the impaired vascular responses in women may have been overcome by the total ischemia induced by the maximal effort in both sexes. However, it is possible to hypothesize that these blunted responses may be present during submaximal walking in women, which deserves investigation since such differences may have implications for tailoring exercise training recommendations for men and women with PAD and IC.

Therefore, the aim of this study was to verify and compare the acute effects of submaximal walking on vascular function, circulating expression of miRNA-126 (c-miRNA-126) and markers of the angiogenic signaling pathway in men and women with PAD and IC.

## Materials and methods

### Ethical aproval

All participants gave free and informed consent before their inclusion in the study, and details that could reveal the identity of the subjects under study have been omitted. The study was approved by the Ethics Committee of Human Research from the University of São Paulo (process 5.307.371). The study was performed in accordance with the ethical standards of the Declaration of Helsinki.

### Study design

This was a randomized controlled trial in which two groups of patients with PAD and IC (men and women) underwent, in a random order (htpps://www.randomizer.org), two experimental sessions: exercise (ES) and control (CS). Participants were evaluated before and after the intervention (exercise or rest, respectively). The main outcomes were blood expression levels of c-miRNA-126, miRNA-126 targets (PI3KR2 and SPRED-1), VEGF and nitric oxide synthase enzyme (eNOS).

### Patients

Patients with PAD and IC were recruited from a vascular center. Inclusion criteria were: (i) aged *≥* 50 years; (ii) diagnosis of PAD at Rutherford stages 1–3 (Gerhard-Herman et al. 2017); (iii) ankle brachial index (ABI) *≤* 0.90; (iv) systolic/diastolic blood pressures (SBP/DBP) *≤* 160/105mmHg; (v) absence of revascularization or angioplasty in the past year; and (vii) ability to walk at least 2 min at 2.0 mph. Exclusion criteria included: (i) presence of diabetic neuropathy; (ii) clinical conditions contraindicating exercise (e.g., severe musculoskeletal disfunctions, uncontrolled chronic diseases such as uncontrolled diabetes or hypertension, instable heart disease, and others); (iii) myocardial ischemia or complex arrhythmias during a maximal walking test; (iv) discontinuation of the maximal walking test for reasons other than IC; and (v) changes in pharmacological treatment during the study period.

### Procedures

Patients were interviewed to identify age, presence of cardiovascular diseases, comorbidity conditions, and current medications. Ankle-brachial index (ABI), body mass index (BMI) and auscultatory BP were measured according to established guidelines (Barroso et al. 2021; Gerhard-Herman et al. 2017), while clinical autonomic neuropathy was assessed in the diabetic patients following the American Diabetes Association’s battery (American Diabetes 2020). Additionally, all patients underwent a graded walking test until the maximal claudication pain. The test was conducted on a treadmill (Imbrasport, ATL, Porto Alegre, Brazil) following a standardized protocol (2 mph, 2% increment every 2 min)(Gardner et al. 1991). During the test, the patients were monitored by an ECG (Welch Allyn, Inc., Cardio Perfect MD, New York, USA), and the heart rate (HR) at the pain threshold (beginning of claudication pain) was recorded (da Rocha Chehuen et al. 2016). Additionally, walking capacity was assessed by the distance walked until the onset of leg pain (claudication onset distance – COD) and the maximal distance walked (total walking distance – TWD).

Male and female patients who fulfilled the study criteria underwent the two experimental sessions (ES and CS) that were performed in a random order with a minimum interval of 3 days between them. The sessions were held in the morning (between 7 a.m. and 12 p.m.) in a temperature-controlled laboratory (20–23 °C). Patients were instructed to fast for at least 8 h before the sessions, and not to perform vigorous exercise nor to drink alcohol in the previous 24 h. In addition, they had to take their regular medications as normal.

In each experimental session, data were collected before and after the specific intervention. For that, upon arrival at the laboratory, the patients laid down, a catheter was inserted into the antecubital vein, and it was kept patent by salinization. Thus, the pre-intervention period started and lasted 40 min. During this period, a blood sample was collected and, subsequently, HR and BP were measured in triplicate, while baseline blood flow (BBF) and FMD were assessed in the brachial arm at the last 20 min. Then, the patients moved to the treadmill and performed the specific intervention of each experimental session (60 min). In the ES, they performed 15 bouts of 2-min walking interspersed by 2-min of upright passive rest. During the walk, the treadmill speed was kept at 2 mph, and the grade was adjusted to maintain the patient´s HR at the HR obtained at the pain threshold (± 4 bpm) assessed at the graded walking test. A previous study showed that the HR at pain threshold corresponds to a metabolic stimulus higher than the anaerobic threshold, revealing a moderate intensity (Ritti-Dias et al. 2009). In the CS, the patients remained standing on the treadmill. After the intervention, the patients immediately returned to supine position and rested for 50 min (post-intervention). During this period, at 20 min, HR and BP were measured in triplicate, followed by a blood sample collection. Then, BBF and FMD were assessed.

### Measurements

#### Blood pressure

BP was measured on the patient’s dominant arm using the auscultatory technique, employing a stethoscope and a mercury column sphygmomanometer, and accepting the phases I and V of the Korotkoff sounds to identify SBP and DBP, respectively. Mean arterial pressure (MBP) was calculated as MBP=(SBP-DBP)/3 + DBP.

#### ABI

SBP was simultaneously measured in the brachial artery and posterior tibial artery using the auscultatory method and a handheld Doppler (Martec, DV 6000, Ribeirão Preto, Brazil), respectively. Measurements were performed on both sides of the body, and the ABI was calculated for each leg by the ratio between the ankle SBP and highest brachial SBP. The lower ABI between the two legs was used for sample characterization.

#### Blood flow and flow-mediated dilation

Blood flow and endothelial function, assessed by FMD, were measured in the brachial artery of the arm with the highest BP. The blood flow velocity was determined by duplex ultrasonography through a linear array (L9-3 RS probe, Logiq E (R7), California, USA) with an insonation angle of 60º. Arterial diameter was determined by an automatic software (QUIPU – Cardiovascular Suite, Pisa, Italy) in recordings lasting 60 s (baseline period). To assess FMD, a cuff was positioned on the forearm just below the cubital fossa and was inflated to 250mmHg for 5 min. After deflation of the cuff, images of the brachial artery and blood flow velocity were recorded for 180s. Blood flow was calculated by multiplying the artery diameter by the mean flow velocity, which was expressed as ml/min. Peak shear stress was measured as peak velocity after deflation of the cuff, which was expressed as s^− 1^. FMD was calculated by the percentage increase in the artery diameter after occlusion in relation to baseline (FMD (%)=[(peak diameter – baseline diameter)/baseline diameter]*100). Vascular conductance (VC) was calculated by dividing MBP by blood flow and was expressed in arbitrary units (a.u.).

#### Whole blood RNA isolation

Total RNA, including miRNA, was isolated using the PAXgene Blood miRNA Kit (Qiagen, Hilden, Germany), following the manufacturer’s instructions. After extraction, the total RNA concentration was quantified using a NanoDrop Spectrophotometer (NanoDrop Technologies, Wilmington, DE) and checked for integrity by EtBr-agarose gel electrophoresis. Samples were immediately frozen at −80 °C according to the manufacturer’s instructions.

#### mRNA and miRNA analysis by real-time polymerase chain reaction

RNA was primed with 0.5 mg/ml oligo(dT) (Fermentas/Thermo Scientific Molecular Biology, Rockford, IL) to generate first-strand DNA. Reverse transcription was performed using Revertaid M-MuLV Reverse Transcriptase (Fermentas/Thermo Scientific Molecular Biology). cDNA for miRNA analysis was synthesized from total RNA using gene-specific primers, according to the TaqMan miRNA Assay protocol (Applied Biosystems, Carlsbad, CA). Real-time quantification of SPRED1, PI3KR2, VEGF, and eNOS mRNA were performed with a SYBRGreen PCR Master Mix, using ABI PRISM 7700 Sequence Detection System (Applied Biosystems). The expression of cyclophilin was measured as an internal control for sample variation in the reverse transcription reaction. Primers were designed using the Primer3 software (http://primer3plus.com/cgi-bin/dev/primer3plus.cgi). The DNA sequence was obtained from GenBank, and primers were made in separate exons to distinguish PCR products derived from cDNA by size from those derived from genomic DNA contaminants. To accurately detect mature miRNA- 126 (INV 2228), the real-time PCR quantification method was performed using TaqMan miRNA Assay Protocol (Applied Biosystems). miRNAs samples were normalized by evaluating U6 expression. Each blood sample was analyzed in duplicate. Relative quantities of target gene expressions were compared after normalization to the values of the reference gene (ΔCT). Fold changes in mRNA and miRNA expression were calculated using the differences in ΔCT values between the two samples (ΔΔCT) and the Eq. 2^−ΔΔCT^. Results are expressed as percentage of CS baseline.

### Statistical analysis

Normality of data distribution and homogeneity of variance were confirmed by the Shapiro-Wilk and Levene tests, respectively. Groups’ characteristics were compared by t-tests or chi-squared tests. For the main outcomes, mixed three-way ANOVAs were employed, establishing group (men and women) as the between main factor, and session (ES and CS) and moment (pre- and post-intervention) as the within main factor. When appropriate, the Bonferroni post-hoc test was used, and *p* ≤ 0.05 was considered as significant (JASP 0.19.3, Amsterdam, Netherlands). Data are presented as means ± SD in the text and tables, while means ± SE were used in the figures for a clearer view.

## Results

Thirty-six patients provided written informed consent to participate in the study, however, 4 were excluded for not fulfilling the inclusion criteria (2 men and 2 women). Thus, 32 patients began the protocol, but 1 man and 1 woman dropped out due to personal reasons. Therefore, 30 patients, 15 in each group, completed the study. The physical characteristics and the severity of PAD as well as the prevalence of medication use and comorbidities were similar between the groups (Table 1).

**Table 1:** Physical characteristics andseverity in men and women with PAD

No.	Men	Women	*p* value
	15	15	
Age – ys	68 ± 5	64 ± 8	0.102
Body mass index - kg/m^2^	26.8 ± 4.1	24.8 ± 4.8	0.235
*Diagnosis of peripheral artery disease*			
Ankle brachial index (worst leg)	0.60 ± 0.15	0.53 ± 0.19	0.359
*Comorbidities (%)*			
Hypertension	80	67	0.682
Current smoker	67	74	0.690
Diabetes mellitus	34	27	0.409
*Medication*			
Acetylsalicylic acid	67	94	0.169
Statin	94	94	1.000
Oral hypoglycemic	34	34	1.000
*Antihypertensive agents*			
Β-blocker	54	34	0.269
Diuretic	40	34	0.705
Dihydropyridine calcium channel antagonist	34	27	0.690
Angiotensin converting enzyme inhibitor	20	27	1.000
Angiotensin II receptor antagonist	54	34	0.269

Results are presented as mean ± standard deviation or percentage (%)

Additionally, walking capacity (COD and TWD) and the HR at the pain threshold were also similar between men and women (331 ± 71 vs. 270 ± 50 m, *p* = 0.551; 624 ± 87 vs. 522 ± 78 m, *p* = 0.603; and 99 ± 4 vs. 102 ± 4 bpm, *p* = 0.704, respectively).

Considering the cardiovascular outcomes, all ANOVAs revealed significance only for the interaction between session and moment (Table 2). Thus, regardless of the group (men or women), from pre- to post-intervention, HR increased significantly in ES and did not change in CS, SBP increased significantly in the CS and decreased in the ES, and DBP increased significantly in CS and did not change in the ES. Consequently, after the interventions, HR was significantly higher while SBP and DBP were significantly lower in the ES compared to the CS.

**Table 2:** Cardiovascular and vascular variables measured at baseline and after sessions in groups of men and women with PAD

		Men			Women			Significance	
		Pre	Post		Pre	Post			
*Cardiovascular function*									
HR (bpm)	CS	65 ± 14	65 ± 15		62 ± 10	60 ± 10		P_session vs. Moment =_**0.006**	
	ES	64 ± 13	67 ± 14*#		64 ± 12	66 ± 13*#			
SBP (mmHg)	CS	137 ± 17	145 ± 13*		140 ± 15	146 ± 15*		P_session vs. Moment =_**<0.001**	
	ES	136 ± 17	128 ± 18*#		138 ± 15	130 ± 17*#			
DBP (mmHg)	CS	81 ± 9	83 ± 8*		78 ± 7	80 ± 6*		P_session vs. Moment =_**<0.001**	
	ES	82 ± 9	80 ± 8**#**		80 ± 6	76 ± 8#			
*Vascular function*									
BBF (ml/min)	CS	133 ± 78	119 ± 69		100 ± 51†	79 ± 31†		P_group =_ **0.001**	
	ES	162 ± 79	150 ± 51		83 ± 42†	92 ± 45†			
VC (ml.min^− 1^.mmHg^− 1^)	CS	1.3 ± 0.8	1.6 ± 2.1		1.0 ± 0.7†	0.8 ± 0.3†		P_group =_ **0.019**	
	ES	1.6 ± 0.8	1.5 ± 1.2		0.8 ± 0.4†	1.0 ± 0.5†			
FMD (%)	CS	6.6 ± 4.2	6.7 ± 2.7		6.0 ± 2.5	5.7 ± 3.2		All *P* > 0.05	
	ES	6.6 ± 2.2	7.7 ± 2.3		7.1 ± 3.9	7.5 ± 4.3			
Baseline diameter (mm)	CS	4.47 ± 0.4	4.52 ± 0.6		3.73 ± 0.5	3.59 ± 0.3		All *P* > 0.05	
	ES	4.49 ± 0.5	4.43 ± 0.5		3.71 ± 0.5	3.66 ± 0.5			
Peak shear stress (s^− 1^)	CS	663 ± 125	612 ± 219		738 ± 158	757 ± 244		All *P* > 0.05	
	ES	684 ± 146	630 ± 175		737 ± 125	824 ± 166			

*Data are presented as mean± standard deviation. HR* :Heart rate; *SBP* :  Systolic blood pressure; *DBP*: Diastolic blood pressure; *BBF* :  Brachial blood flow; *VC* : Vascular conductance; *FMD* : Flow mediated dilation. Bold = significant result. * Significantly different from pre at the same session (p *≤* 0.05); # Significantly different from the control session at the same moment (p *≤* 0.05); † Significantly different from men at the same session and moment (p *≤* 0.05)

Regarding the vascular outcomes, ANOVAs revealed only a significant group effect for BBF and VC (Table 2), showing that BBF and VC were greater in men than women, regardless of the session and the moment, and they did not change significantly from pre- to post-intervention in any of the sessions. Additionally, no significant effects or interactions were detected for FMD, baseline arterial diameter and peak shear stress (Table 2), indicating that these variables were similar between the genders and did not change significantly in any session.

**Fig. 1:**
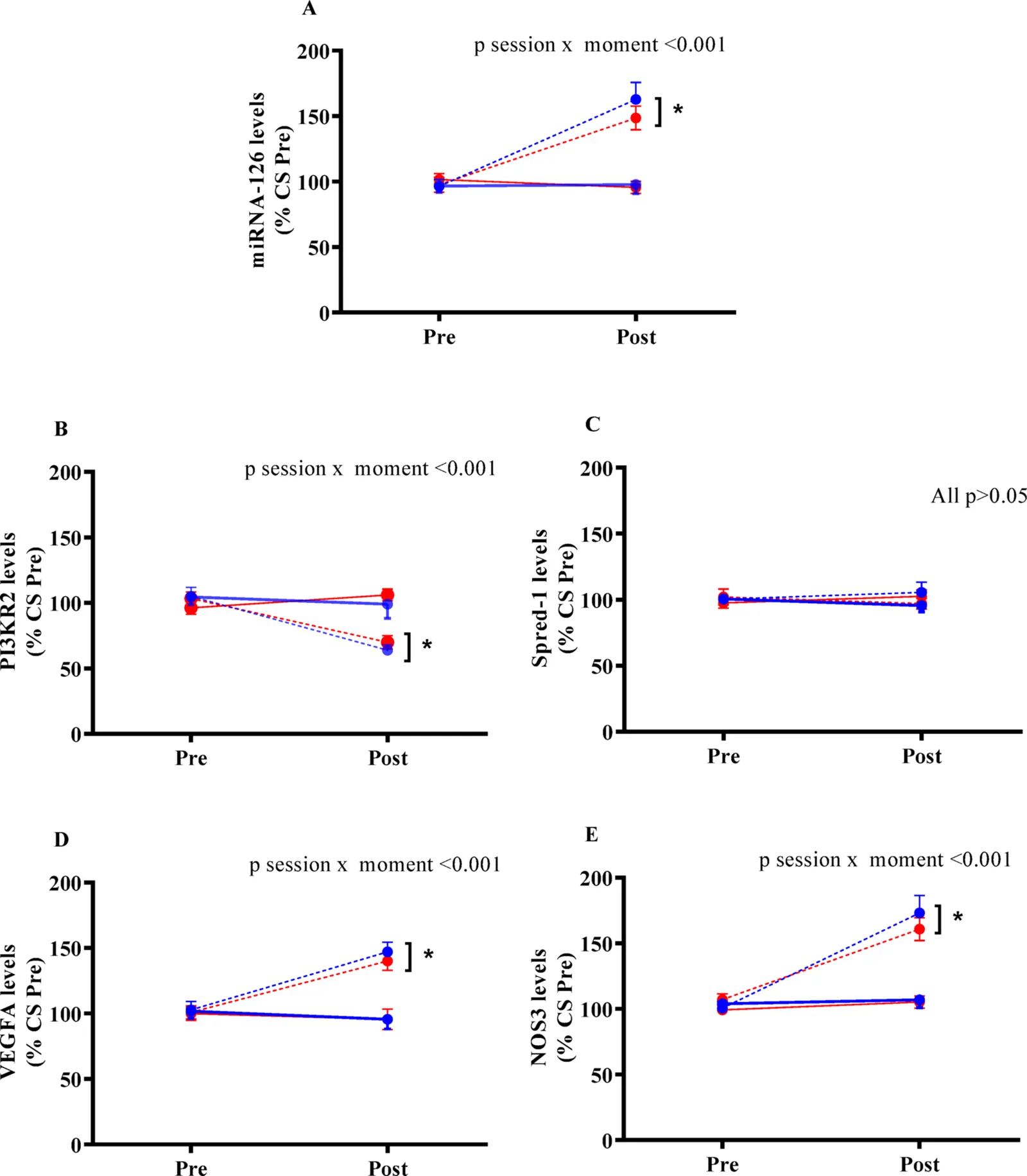
Circulation expression of microRNA-126 (miRNA-126, panel A), vascular endothelial growth factor (PI3KR2, panel B); sprouty-related protein 1 (SPRED-1, panel C); phosphoinositol-2 kinase regulatory subunit 2 (VEGF, panel D) and nitric oxide synthase (eNOS, panel E) assessed at baseline and after sessions for groups of men (*n* = 15, blue line) and women (*n* = 15, red line), dashed line (exercise session), solid line (control session). Data: mean ± SE. * different from baseline within the group (*p* < 0.05)

Also, for all blood markers, ANOVAs only detected significant interactions between session and moment (Fig. 1). Thus, c-miRNA-126 increased significantly and similarly from pre- to post-intervention in the ES and did not change in the CS in both groups (Men, CS = 96.5 ± 4.7 vs. 97.8 ± 6.9% and ES = 96.4 ± 5.4 vs. 162.7 ± 13.0%, Women, CS = 101.5 ± 4.5 vs. 95.6 ± 4.7% and ES = 97.5 ± 5.7 vs. 148.5 ± 9.0%, p_session x moment_ <0.001). The expression of PI3KR2 decreased significantly from pre- to post-intervention in the ES and did not change in the CS in both groups (Men, CS = 104.5 ± 5.7 vs. 99.0 ± 10.8% and ES = 105.1 ± 6.8 vs. 63.9 ± 4.1%, Women, CS = 96.3 ± 4.9% vs. 106.0 ± 4.6 and ES = 103.4 ± 5.0 vs. 69.8 ± 5.2%, p_session x moment_ <0.001). The expression of Spred-1 did not change significantly in any session or group (Men, CS = 100.5 ± 3.3 vs. 95.5 ± 4.8% and ES = 100.4 ± 3.7 vs. 105.6 ± 7.7%, Women, CS = 97.6 ± 4.1 vs. 102.6 ± 5.0% and ES = 102.1 ± 6.1 vs. 97.1 ± 4.0%, p_session x moment_ =0.433). Finally, the expression of VEGF and eNOS increased significantly and similarly from pre- to post-intervention in the ES and did not change in CS in both groups (VEGF - Men, CS = 101.7 ± 5.4 vs. 95.6 ± 7.3% and ES = 102.9 ± 6.5 vs. 147.1 ± 7.2%, Women, CS = 100.0 ± 5.1 vs. 95.0 ± 7.9% and ES = 101.0 ± 5.0 vs. 140.0 ± 7.3%, p_session x moment_ <0.001; and eNOS - Men, CS = 104.0 ± 4.7 vs. 107.0 ± 6.0% and ES = 107.0 ± 4.4 vs. 173.1 ± 13.3%, Women, CS = 99.2 ± 3.2 vs. 105.3 ± 4.7% and ES = 99.2 ± 3.2 vs. 160.8 ± 8.8%, p_session x moment_ <0.001).

## Discussion

The main findings of this study were that acute SW induced a significant increase in c-miRNA-126 and VEGF, thereby enhancing the PI3KR2 - eNOS angiogenic pathway. Contrary to the initial hypothesis, these responses were similar in men and women with PAD and IC.

In the present study, there was no difference in walking capacity (assessed either by COD or TWD) between the genders, which is similar to a previous study from our group (Miyasato et al. 2021), but differs from other studies (Gardner et al. 2009; Gardner 2002) that have reported lower walking capacity in women than men with PAD using the same test protocol. Nevertheless, as in our previous study (Miyasato et al. 2021), this study employed careful participation criteria to ensure good matching between the genders regarding their physical and clinical characteristics (Table I). This match was essential to ensure that any post-exercise difference between men and women can be attributed to gender and not to physical or clinical differences. In general, previous studies have matched the genders groups only by ABI and age, which may have contributed to the differences found between genders, since the presence of factors, such as comorbidities presence, may affect walking capacity in patients with PAD (Gardner et al. 2016).

Similarly to the walking capacity results, BP responses following submaximal walking also did not differ between the genders. Post-walking SBP and DBP were significantly lower in the ES compared to the CS, revealing similar post-exercise hypotension in men and women with PAD. This result is in accordance with studies that have reported significant BP reduction after acute walking in patients with PAD and IC (Cucato et al. 2015; Chehuen et al. 2021) and with our previous study that showed similar post-exercise hypotension in male and female patients after maximal walking (Miyasato et al. 2021), but it expands the knowledge by showing that BP reduction after walking is not affected by gender even after the submaximal effort that is usually recommended for this population.

Regarding the vascular function, submaximal walking did not significantly alter any of the variables studied (BBF, VC and FMD) in either men or women with PAD. Differently, walking to maximum intensity have been reported to increase BBF (da Silva et al. 2015; Joras and Poredos 2008) and reduce FMD (Joras and Poredos 2008; Silvestro et al. 2002), and the discrepancy between the results may be explained by the fact that maximal effort generates greater ischemia than the exercise employed in this study. However, chronically, submaximal walking training has been shown to increase FMD (Januszek et al. 2014), muscle capillarization and angiogenic markers (da Silva et al. 2022) besides improving walking capacity. Therefore, despite the lack of acute post-walking changes, the long-term vascular adaptations obtained with submaximal walking are beneficial to this population.

Although SW did not promote acute changes in post-exercise vascular function, it similarly modified of the c-miRNA-126, its target PI3KR2, and the angiogenic markers, VEGF and eNOS, in men and women with PAD. It is known that the expression of c-miRNA-126 is markedly reduced in patients with PAD (Stather et al. 2013), which may be, in part, responsible for releasing the negative regulator PIK3R2 pathway, resulting in reduced VEGF and eNOS in this population (Fish et al. 2008). The current study showed, for the first time, that submaximal walking is able to acutely increase c-miRNA-126, decrease PIK3R2, and consequently increase VEGF and eNOS expressions in whole blood, which suggest that this type of exercise may be a trigger for the activation of this important angiogenic pathway that is reduced in patients with PAD. These results corroborate previous studies by our group that have showed the same changes in this signaling pathway in men with PAD after maximal walking (da Silva et al. 2015) and after a period of walking training (da Silva et al. 2022).

The post-exercise responses of c-miRNA-126 expression and the angiogenesis pathway were not different between the men and women with PAD and IC. This similarity occurred with a submaximal walking performed at the same absolute and relative intensities since men and women presented similar maximal walking capacities. Thus, differently than hypothesized, these findings support that, when gender groups are paired by clinical characteristics and functional capacity, women with PAD do not present compromised vascular responses to exercise even during a submaximal effort that do not impose total ischemia. Therefore, the worse vascular function previously reported in literature for women with PAD may be related to their worse functional capacity and not to the gender per se, which should be checked in the future by researchers with specific designs for this comparison. In addition, the fact that acute submaximal walking was equally effective in inducing changes in the molecular angiogenic pathways that may chronically promote vascular benefits in PAD, support that women and men with PAD and IC may equally respond to walking training.

Therefore, the present study revealed two new findings that have clinical relevance. First, it showed that, like maximal walking, acute submaximal walking can stimulate angiogenic pathways in patients with PAD and IC. Second, it revealed that improvements induced by acute walking are similar in men and women with PAD and IC. Together, these findings give scientific support for the recommendation of submaximal walking training for both men and women with PAD and IC as already done in current guidelines (Hirsch et al. 2006).

The current study presents some limitations. First, its results are limited to patients who met the specific inclusion criteria. The length of disease was not assessed, but all patients, men and women, had similar disease severity and walking limitations. Thus, comparison between genders may produce different results if men and women present baseline differences in physical characteristics, clinical status or walking capacity. Additionally, the experiments were conducted with the patients in the fasting state. As the fasting period was similar before both sessions and in both groups, it may not have affected the present results. Finally, it is interesting to note that the study compared the acute effects of submaximal walking, and the chronic effects of walking training may be different, which should be further investigated.

## Abbreviations

ABI: Ankle brachial index
ANOVA: Analysis of variance
BBF: Baseline brachial blood flow
BMI: Body mass index
COD: Claudication onset distance
CS: Control session
c-miRNA-126: Circulating expression of microRNA-126
DBP: Diastolic blood pressure
eNOS: Nitric oxide synthase enzyme
IC: Intermittent claudication
ES: Exercise session
FMD: Flow-mediated dilation
HR: Heart rate
MBP: Mean blood pressure
PAD: Peripheral artery disease
PI3KR2: Phosphoinositol-2 kinase regulatory subunit 2
SBP: Systolic blood pressure
SPRED-1: Sprouty-related protein 1
SW: Submaximal walking
TWD: Total walking distance
VC: Vascular conductance
VEGF: Vascular endothelium growth factor

## Declarations

### Data Availability

The data supporting the findings of this study are not publicly available because participants did not provide consent for unrestricted data sharing. De-identified data are available from the corresponding author upon reasonable request, subject to ethical and institutional approval where applicable.

## References

[CR1] American Diabetes, Association (2020) 11. Microvascular complications and foot care: standards of medical care in diabetes-2020. Diabetes Care 43:S135–S51 10.2337/dc20-S011 31862754

[CR2] Askew CD, Green S, Walker PJ, Kerr GK, Green AA, Williams AD, Febbraio MA (2005) Skeletal muscle phenotype is associated with exercise tolerance in patients with peripheral arterial disease. J Vasc Surg 41:802–807 10.1016/j.jvs.2005.01.037 15886664

[CR3] Barroso WKS, Rodrigues CIS, Bortolotto LA, Mota-Gomes MA, Brandao AA, Feitosa ADM, Machado CA, Poli-de-Figueiredo CE, Amodeo C, Mion Junior D, Barbosa ECD, Nobre F, Guimaraes ICB, Vilela-Martin JF, Yugar-Toledo JC, Magalhaes MEC, Neves MFT, Jardim P, Miranda RD, Rmds Povoa SC, Fuchs A, Alessi AJG, Lucena A, Avezum ALL, Sousa A, Pio-Abreu AC, Sposito AMG, Pierin AMG, Paiva ACS, Spinelli ADR, Nogueira N, Dinamarco B, Eibel CLM, Forjaz CRO, Zanini CB, Souza D, Souza EAF, Nilson EFA, Costa EV, Freitas EDR, Duarte ES, Muxfeldt E, Lima Junior EMG, Campana EJ, Cesarino F, Marques F, Argenta FM, Consolim-Colombo FS, Baptista FA, Almeida FAO, Borelli FD, Fuchs FL, Plavnik GF, Salles GS, Feitosa GVD, Silva GM, Guerra H, Moreno Junior HC, Finimundi IC, Back JB, Oliveira Filho JR, Gemelli JG, Mill JM, Ribeiro LAD, Lotaif LSD, Costa L, Magalhaes LF, Drager LC, Martin LCN, Scala MQ, Almeida MMG, Gowdak M, Klein MVB, Malachias MCC, Kuschnir ME, Pinheiro MHE, Borba OM, Filho O, Passarelli Junior OR, Coelho PVO, Vitorino RM, Ribeiro Junior R, Esporcatte R, Franco R, Pedrosa RA, Mulinari RB, Paula RTP, Okawa RF, Rosa SLD, Amaral SR, Ferreira-Filho (2021) Brazilian guidelines of hypertension--2020. Arq Bras Cardiol 116:516–658 10.36660/abc.20201238 PMC9949730 33909761

[CR4] Becker GJ, McClenny TE, Kovacs ME, Raabe RD, Katzen BT (2002) The importance of increasing public and physician awareness of peripheral arterial disease. J Vasc Interv Radiol 13:7–11 10.1016/s1051-0443(07)60002-5 11788688

[CR5] Boger RH, Bode-Boger SM, Thiele W, Junker W, Alexander K, Frolich JC (1997) Biochemical evidence for impaired nitric oxide synthesis in patients with peripheral arterial occlusive disease. Circulation 95:2068–2074 10.1161/01.cir.95.8.2068 9133517

[CR6] Breek JC, Hamming JF, De Vries J, Aquarius AE, van Berge DP Henegouwen (2001) Quality of life in patients with intermittent claudication using the World Health Organisation (WHO) questionnaire. Eur J Vasc Endovasc Surg 21:118–122 10.1053/ejvs.2001.1305 11237783

[CR7] Chehuen M, Andrade-Lima A, Silva Junior N, Miyasato R, Souza RWA, Leicht A, Brum PC, Oliveira EM, Wolosker N, Forjaz CLM (2021) Physiological Responses to Maximal and Submaximal Walking in Patients with Symptomatic Peripheral Artery Disease. Arq Bras Cardiol 117:309–316 10.36660/abc.20200156 PMC8395785 34495225

[CR8] Clyne CA, Mears H, Weller RO, O’Donnell TF (1985) Calf muscle adaptation to peripheral vascular disease. Cardiovasc Res 19:507–12 10.1093/cvr/19.8.507 4042117

[CR9] Criqui MH, Vargas V, Denenberg JO, Ho E, Allison M, Langer RD, Gamst A, Bundens WP, Fronek A (2005) Ethnicity and peripheral arterial disease: the San Diego Population Study. Circulation 112:2703–2707 10.1161/CIRCULATIONAHA.105.546507 16246968

[CR10] Cucato GG, Chehuen Mda R, Ritti-Dias RM, Carvalho CR, Wolosker N, Saxton JM, Forjaz CL (2015) Post-walking exercise hypotension in patients with intermittent claudication. Med Sci Sports Exerc 47:460–467 10.1249/MSS.0000000000000450 25033263

[CR11] da Rocha Chehuen M, Cucato GG, Saes GF, Costa LA, Leicht AS, Ritti-Dias RM, Wolosker N (2016) Reproducibility of Anaerobic and Pain Thresholds in Male Patients With Intermittent Claudication. J Cardiopulm Rehabil Prev 36(5):358–67 10.1097/HCR.0000000000000173 26959497

[CR12] da Silva ND Jr., Roseguini BT, Chehuen M, Fernandes T, Mota GF, Martin PK, Han SW, Forjaz CL, Wolosker N, de Oliveira EM (2015) Effects of oral N-acetylcysteine on walking capacity, leg reactive hyperemia, and inflammatory and angiogenic mediators in patients with intermittent claudication. Am J Physiol Heart Circ Physiol 309:H897-905 10.1152/ajpheart.00158.2015 26116711

[CR13] da Silva ND Jr., Andrade-Lima A, Chehuen MR, Leicht AS, Brum PC, Oliveira EM, Wolosker N, Pelozin BRA, Fernandes T, Forjaz CLM (2022) Walking training increases microRNA-126 expression and muscle capillarization in patients with peripheral artery disease. Genes (Basel). 10.3390/genes14010101 PMC9858623 36672843

[CR14] Fish JE, Santoro MM, Morton SU, Yu S, Yeh RF, Wythe JD, Ivey KN, Bruneau BG, Stainier DY, Srivastava D (2008) miR-126 regulates angiogenic signaling and vascular integrity. Dev Cell 15:272–284 10.1016/j.devcel.2008.07.008 PMC2604134 18694566

[CR15] Gardner AW (2002) Sex differences in claudication pain in subjects with peripheral arterial disease. Med Sci Sports Exerc 34:1695–1698 10.1097/00005768-200211000-00001 12439070

[CR16] Gardner AW, Skinner JS, Cantwell BW, Smith LK (1991) Progressive vs single-stage treadmill tests for evaluation of claudication. Med Sci Sports Exerc 23:402–408 2056896

[CR17] Gardner AW, Parker DE, Montgomery PS, Blevins SM, Nael R, Afaq A (2009) Sex differences in calf muscle hemoglobin oxygen saturation in patients with intermittent claudication. J Vasc Surg 50:77–82 10.1016/j.jvs.2008.12.065 PMC2765784 19223136

[CR18] Gardner AW, Montgomery PS, Blevins SM, Parker DE (2010) Gender and ethnic differences in arterial compliance in patients with intermittent claudication. J Vasc Surg 51:610–615 10.1016/j.jvs.2009.09.059 PMC3033213 20206806

[CR19] Gardner AW, Parker DE, Montgomery PS (2016) Predictors of Improved Walking after a Supervised Walking Exercise Program in Men and Women with Peripheral Artery Disease. Int J Vasc Med 2016(1):2191350 10.1155/2016/2191350 PMC5220567 28116164

[CR20] Gerhard-Herman MD, Gornik HL, Barrett C, Barshes NR, Corriere MA, Drachman DE, Fleisher LA, Fowkes FGR, Hamburg NM, Kinlay S, Lookstein R, Misra S, Mureebe L, Olin JW, Patel RAG, Regensteiner JG, Schanzer A, Shishehbor MH, Stewart KJ, Treat-Jacobson D, Walsh ME (2017) 2016 AHA/ACC Guideline on the Management of Patients With Lower Extremity Peripheral Artery Disease: Executive Summary: A Report of the American College of Cardiology/American Heart Association Task Force on Clinical Practice Guidelines. J Am Coll Cardiol 69:1465–1508 10.1016/j.jacc.2016.11.008 27851991

[CR21] Gommans LN, Scheltinga MR, van Sambeek MR, Maas AH, Bendermacher BL, Teijink JA (2015) Gender differences following supervised exercise therapy in patients with intermittent claudication. J Vasc Surg 62:681–688 10.1016/j.jvs.2015.03.076 26304482

[CR22] Harris LM, Faggioli GL, Shah R, Koerner N, Lillis L, Dandona P, Izzo JL, Snyder B, Ricotta JJ (1995) Vascular reactivity in patients with peripheral vascular disease. Am J Cardiol 76:207–212 10.1016/s0002-9149(99)80066-6 7611168

[CR23] Hirsch AT, Haskal ZJ, Hertzer NR, Bakal CW, Creager MA, Halperin JL, Hiratzka LF, Murphy WR, Olin JW, Puschett JB, Rosenfield KA, Sacks D, Stanley JC, Taylor LM Jr., White CJ, White J, White RA, Antman EM, Smith SC Jr., Adams CD, Anderson JL, Faxon DP, Fuster V, Gibbons RJ, Hunt SA, Jacobs AK, Nishimura R, Ornato JP, Page RL, Riegel B (2006) Practice Guidelines for the management of patients with peripheral arterial disease (lower extremity, renal, mesenteric, and abdominal aortic): a collaborative report from the American Association for Vascular Surgery/Society for Vascular Surgery, Society for Cardiovascular Angiography and Interventions, Society for Vascular Medicine and Biology, Society of Interventional Radiology, and the ACC/AHA Task Force on Practice Guidelines (Writing Committee to Develop Guidelines for the Management of Patients With Peripheral Arterial Disease): endorsed by the American Association of Cardiovascular and Pulmonary Rehabilitation; National Heart, Lung, and Blood Institute; Society for Vascular Nursing; TransAtlantic Inter-Society Consensus; and Vascular Disease Foundation. Circulation 113(11):463–654 10.1161/CIRCULATIONAHA.106.174526 16549646

[CR24] Januszek R, Mika P, Konik A, Petriczek T, Nowobilski R, Nizankowski R (2014) Effect of treadmill training on endothelial function and walking abilities in patients with peripheral arterial disease. J Cardiol 64:145–151 10.1016/j.jjcc.2013.12.002 24438856

[CR25] Joras M, Poredos P (2008) The association of acute exercise-induced ischaemia with systemic vasodilator function in patients with peripheral arterial disease. Vasc Med 13:255–62 10.1177/1358863X08096347 18940901

[CR26] Kroger K, Stang A, Kondratieva J, Moebus S, Beck E, Schmermund A, Mohlenkamp S, Dragano N, Siegrist J, Jockel KH, Erbel R, Group Heinz Nixdorf Recall Study (2006) Prevalence of peripheral arterial disease - results of the Heinz Nixdorf recall study. Eur J Epidemiol 21:279–85 10.1007/s10654-006-0015-9 16685578

[CR27] Miyasato RS, Felix AJ, Andrade-Lima A, da Silva ND Jr., Ritti-Dias RM, Wolosker N, Cornelissen V, Goessler KF, Forjaz CLM (2021) Cardiovascular responses during and after maximal walking in men and women with symptomatic peripheral artery disease. Ann Vasc Surg 71:9–18 10.1016/j.avsg.2020.07.051 32800891

[CR28] Myers SA, Johanning JM, Stergiou N, Lynch TG, Longo GM, Pipinos II (2008) Claudication distances and the Walking Impairment Questionnaire best describe the ambulatory limitations in patients with symptomatic peripheral arterial disease. J Vasc Surg 47:550–555 10.1016/j.jvs.2007.10.052 18207355

[CR29] Norgren L, Hiatt WR, Dormandy JA, Nehler MR, Harris KA, Fowkes FG, Tasc Ii Working Group (2007) Inter-society consensus for the management of peripheral arterial disease (TASC II). J Vasc Surg 45(Suppl S):S5-67 10.1016/j.jvs.2006.12.037 17223489

[CR30] Palmer-Kazen U, Wariaro D, Luo F, Wahlberg E (2004) Vascular endothelial cell growth factor and fibroblast growth factor 2 expression in patients with critical limb ischemia. J Vasc Surg 39:621–8 10.1016/j.jvs.2003.07.006 14981457

[CR31] Ritti-Dias RM, de Moraes Forjaz CL, Cucato GG, Costa LA, Wolosker N, Fatima Nunes Marucci (2009) Pain threshold is achieved at intensity above anaerobic threshold in patients with intermittent claudication. J Cardiopulm Rehabil Prev 29:396–401de 10.1097/HCR.0b013e3181b4ca38 19770807

[CR32] Sigvant B, Wiberg-Hedman K, Bergqvist D, Rolandsson O, Andersson B, Persson E, Wahlberg E (2007) A population-based study of peripheral arterial disease prevalence with special focus on critical limb ischemia and sex differences. J Vasc Surg 45:1185–1191 10.1016/j.jvs.2007.02.004 17543683

[CR33] Silvestro A, Scopacasa F, Oliva G, de Cristofaro T, Iuliano L, Brevetti G (2002) Vitamin C prevents endothelial dysfunction induced by acute exercise in patients with intermittent claudication. Atherosclerosis 165:277–83 10.1016/s0021-9150(02)00235-6 12417278

[CR34] Song P, Rudan D, Zhu Y, Fowkes FJI, Rahimi K, Fowkes FGR, Rudan I (2019) Global, regional, and national prevalence and risk factors for peripheral artery disease in 2015: an updated systematic review and analysis. Lancet Glob Health 7:e1020–e30 10.1016/S2214-109X(19)30255-4 31303293

[CR35] Stather PW, Sylvius N, Wild JB, Choke E, Sayers RD, Bown MJ (2013) Differential microRNA expression profiles in peripheral arterial disease. Circ Cardiovasc Genet 6:490–497 10.1161/circgenetics.111.000053 24129592

[CR36] Vita JA, Hamburg NM (2010) Does endothelial dysfunction contribute to the clinical status of patients with peripheral arterial disease? Can J Cardiol 26(Suppl A):45A–50A 10.1016/s0828-282x(10)71062-x 20386761

